# *BRIP1*, *RAD51C*, and *RAD51D* mutations are associated with high susceptibility to ovarian cancer: mutation prevalence and precise risk estimates based on a pooled analysis of ~30,000 cases

**DOI:** 10.1186/s13048-020-00654-3

**Published:** 2020-05-02

**Authors:** Malwina Suszynska, Magdalena Ratajska, Piotr Kozlowski

**Affiliations:** 1 Department of Molecular Genetics, Institute of Bioorganic Chemistry, Polish Academy of Sciences, Noskowskiego 12/14 Street, 61-704 Poznan, Poland; 2 Department of Pathology, Dunedin School of Medicine, University of Otago, 60 Hanover Street, Dunedin, 9016 New Zealand; 3 Department of Biology and Medical Genetics, Medical University of Gdansk, Debinki 1 St., 80-210 Gdansk, Poland

**Keywords:** *BRIP1*, *RAD51C*, *RAD51D*, Meta-analysis, Ovarian cancer risk

## Abstract

**Background:**

It is estimated that more than 20% of ovarian cancer cases are associated with a genetic predisposition that is only partially explained by germline mutations in the *BRCA1* and *BRCA2* genes. Recently, several pieces of evidence showed that mutations in three genes involved in the homologous recombination DNA repair pathway, i.e., *BRIP1*, *RAD51C*, and *RAD51D*, are associated with a high risk of ovarian cancer. To more precisely estimate the ovarian cancer risk attributed to *BRIP1*, *RAD51C*, and *RAD51D* mutations, we performed a meta-analysis based on a comparison of a total of ~ 29,400 ovarian cancer patients from 63 studies and a total of ~ 116,000 controls from the gnomAD database.

**Results:**

The analysis allowed precise estimation of ovarian cancer risks attributed to mutations in *BRIP1*, *RAD51C*, and *RAD51D*, confirming that all three genes are ovarian cancer high-risk genes (odds ratio (OR) = 4.94, 95%CIs:4.07–6.00, *p* < 0.0001; OR = 5.59, 95%CIs:4.42–7.07, *p* < 0.0001; and OR = 6.94, 95%CIs:5.10–9.44, *p* < 0.0001, respectively). In the present report, we show, for the first time, a mutation-specific risk analysis associated with distinct, recurrent, mutations in the genes.

**Conclusions:**

The meta-analysis provides evidence supporting the pathogenicity of *BRIP1*, *RAD51C*, and *RAD51D* mutations in relation to ovarian cancer. The level of ovarian cancer risk conferred by these mutations is relatively high, indicating that after *BRCA1* and *BRCA2*, the *BRIP1*, *RAD51C*, and *RAD51D* genes are the most important ovarian cancer risk genes, cumulatively contributing to ~ 2% of ovarian cancer cases. The inclusion of the genes into routine diagnostic tests may influence both the prevention and the potential treatment of ovarian cancer.

## Background

Ovarian cancer (OC) is the cause of over 180,000 deaths among women worldwide each year [1]. Due to the asymptomatic nature of OC and the absence of effective screening tests for detecting the early stage of the disease, approximately 70% of women are diagnosed at an advanced stage. Late-stage diagnosis of OC results in a 5-year relative survival rate of 29%, in contrast with 92% for early-stage disease [2]. Considering the increased survival rate associated with the early detection of OC, a prevention program employing genetic and epidemiological risk factor status to identify women at high OC risk, followed by relevant lifestyle and surgical strategies, could substantially decrease the number of OC-related deaths. A strategy applying preventive genetic testing seems to be very attractive, especially for OC, as more than 20% of women diagnosed with OC have a hereditary tendency to develop the disease, harboring a loss-of-function mutation in one of the already known cancer-related genes [3, 4]. Owing to the development of advanced high-throughput sequencing technologies and clinical genetic testing, these mutations will likely be easier to identify in a larger population in the near future.

Most of the identified germline mutations in OC patients occur in the highly penetrant genes *BRCA1* and *BRCA2* (*BRCA1*/*2*), whose proteins are involved in fundamental cellular processes, including DNA repair [5–7]. Mutations in *BRCA1*/*2* genes result in homologous recombination (HR) deficiency which may be utilized in the treatment of OC with platinum-based chemotherapy and poly ADP-ribose polymerase (PARP) inhibitors [6, 8–10]. Other mechanisms of HR deficiency, which lead to phenotype described as BRCAness, include germline and somatic mutations in other HR-related genes, epigenetic modifications (e.g., *BRCA1, RAD51C* promoter hypermethylation) [11–13], and *EMSY* amplification/overexpression [14, 15]. Even up to 50% of OC exhibit HR deficiency (most commonly high-grade serous OC), therefore a substantial fraction of OC patients may benefit from therapeutic approaches based on PARP inhibitors [16].

Among other HR-related genes whose association with OC risk has been recently well documented are *BRCA1-interacting protein C-terminal helicase 1* (*BRIP1*, *also known as BACH1* or *FANCJ*) [17, 18], *RAD51 homolog C* (*RAD51C*) [19, 20], and *RAD51 paralog D* (*RAD51D*) [21, 22], coding for proteins that interact with BRCA1/2 and support the DNA repair process. Patients with germline mutations in *BRIP1*, *RAD51C*, and *RAD51D*, could likely also benefit from therapy with PARP inhibitors [21, 23]. At present, it is recommended for *BRIP1*, *RAD51C*, and *RAD51D* mutation carriers beginning at age 45–50 to consider risk-reducing salpingo-oophorectomy [24].

The results of several recent studies suggest that after *BRCA1* and *BRCA2*, the *BRIP1*, *RAD51C*, and *RAD51D* genes may be the most important OC predisposition genes. Cumulatively, germline mutations in *BRIP1*, *RAD51C*, and *RAD51D* account for ~ 2% of OC cases [25, 26], and they seem to be predominantly associated with the high risk of OC [18, 25–29], in contrast to mutations in other common genes (including *BRCA1*/*2*, *TP53*, *PTEN,* and the mismatch repair *MSH2* and *MSH6* genes), contributing also to breast cancer (BC). The estimated cumulative OC risk is 5.8, 5.2, and 12% for *BRIP1* [18] (by age 80), *RAD51C*, and *RAD51D* (by age 70) [28] mutation carriers, respectively.

Although the evidence is strong, the risk attributed to particular genes varies substantially among studies (odds ratio (OR) values estimated for *BRIP1, RAD51C, and RAD51D* range ~ 5–19, ~ 5–15, and of ~ 6–12, respectively, for mutations) [18, 26–29]. The unreliability of risk estimation results mostly from the following factors: (i) the mutation prevalence within the discussed genes is generally low (up to ~ 1% among unselected OC patients); (ii) individual OC studies are not common (in comparison to BC studies) and usually encompass a limited number of cases, therefore more extensive sample sizes are needed to precisely determine the associations; (iii) most studies do not carry out the analysis of the equivalent control group, hindering the interpretation of results; (iv) studies often enroll affected probands with either BC or OC from BC and/or OC families, demonstrating collective results that do not allow distinguishing cancer-specific risks; and (v) the risk estimates may be affected by different effects of individual mutations over- or underrepresented in particular populations/studies.

Therefore, to more precisely establish the risk estimates, we performed a cumulative analysis of already published epidemiological studies that analyzed the *BRIP1*, *RAD51C*, and *RAD51D* genes of patients exclusively with OC. Taking advantage of 443 mutations from 63 studies [18, 21, 26–86], encompassing a total of ~ 29,400 OC patients, we determined with high confidence the OC risk associated with all mutations within the discussed genes. As both the mutation location and its effect on encoded protein may influence its pathogenicity, therefore, for all recurrent mutations specified in this study, we calculated the mutation-specific risk. It was the first attempt to estimate mutation-specific OC risk for a wide spectrum of recurrent mutations in *BRIP1*, *RAD51C*, or *RAD51D*.

The results of our analysis provide stronger evidence for the pathogenic role of *BRIP1*, *RAD51C*, and *RAD51D* mutations, and may be utilized in establishing guidelines for OC prevention and therapeutic strategies for carriers.

## Methods

The relevant papers that reported results of the sequence analysis of *BRIP1*, *RAD51C*, and/or *RAD51D* genes in OC cases, published before September 2019, were searched from the PubMed electronic database using the combined terms of “ovarian cancer”, “BRIP1/BACH1/FANCJ”, “RAD51C”, “RAD51D”, “multi-gene/multigene panel”, “whole exome sequencing”, “germline”, “risk” and “mutation”. Studies encompassing OC patients unselected for family history and familial OC cases (also extracted from both BC and OC familial studies) and multicancer studies, including patients with either OC and BC, were taken into consideration. Also, patients with all reported histological subtypes of OC were included. Studies were excluded if they provided insufficient data for extraction, i.e., the number of OC subjects, a list of identified variants, or the type of cancer assigned for a particular variant. Additionally, case reports and common sequence variant studies were not included. The search was restricted to germline, definitive loss-of-function variants (i.e., frameshift, nonsense, ±1/±2 position splicing mutations) and missense and intron variants described as pathogenic/likely pathogenic in the ClinVar database [87, 88], identified by the whole sequence of gene analysis. No minimum coverage of targets was specified for next-generation sequencing (NGS) study inclusion. To maximize the accuracy of the study, the screening of eligible studies and data extraction was performed by two reviewers (MS and PK).

As the mutation frequencies in the genes investigated in this study are relatively small, all ethnicities/races were included to collect a maximum number of results from populations that underwent genetic testing, which makes the study more extensive and comprehensive. The cumulative mutation frequency and the frequency of each pathogenic variant were calculated by pooling data (the number of variants/the number of analyzed OC patients) from the selected studies. The overall (gene-specific) and mutation-specific OC risks were estimated by a comparison of the mutation frequencies in OC patients to the overall and particular mutation frequencies in controls, extracted from the online non-cancer Genome Aggregation Database (gnomAD) [89–91] as of July 2019. Subjects whose DNA sequencing data are placed within the non-cancer gnomAD databases are free of cancer; however, the family history of cancer is unknown. Taking the place of origin into account, the adjusted risk was estimated using major populations (i.e., Caucasian and East Asian) and appropriate population controls from the non-cancer gnomAD database.

Associations between mutations in investigated genes and OC risk were assessed using odds ratios (ORs) and 95% confidence intervals (95% Cls) based on a chi-squared test. Adjusted OR (OR_adj_) was estimated with the use of the logistic regression model. All statistical tests were two-sided, and a *p*-value of less than 0.05 was considered statistically significant. MedCalc Statistical Software version 14.8.1 (MedCalc Software bvba, Ostend, Belgium; http://www.medcalc.org) was used for all analyses.

## Results

To precisely estimate the OC risk associated with mutations in *BRIP1*, *RAD51C*, and *RAD51D*, we searched for studies that provided information on mutation frequency in the abovementioned genes in OC cases and then compared these data to the mutation frequencies in population controls from the publicly available gnomAD database. The initial PubMed database search identified 1062 records; 925 unrelated records were removed based on title/abstract review, then 78 articles, not meeting the criteria (described in the Methods section), were removed based on the full-text review (Figure S1). Fifty nine articles fulfilled the inclusion criteria and were included in this analysis, along with 4 hand-searched articles. Separately, the selection criteria were met by 44, 53, and 42 studies analyzing the whole coding sequence of *BRIP1*, *RAD51C*, and *RAD51D*, respectively. In 41 out of 63 studies, NGS of either multigene panels or whole-exome sequencing was applied to identify mutations. In the remaining (mostly older) studies, mutations were identified with the use of high resolution melting (HRM)/ denaturing gradient gel electrophoresis (DGGE)/ denaturing high-performance liquid chromatography (DHPLC) and then sequencing or directly by Sanger sequencing. The number of patients with OC participating in individual studies ranged from several to several thousand, with five studies comprising ~ 2000 or more patients [18, 26, 28, 29, 85]. Overall, 443 mutations were identified in the discussed genes in 29,382 cases in 63 studies, giving a combined mutation frequency of 1.93%. Generally, an association between mutations in all three genes and susceptibility to OC at the level of OR > 4 has been observed.

**Fig. 1:**
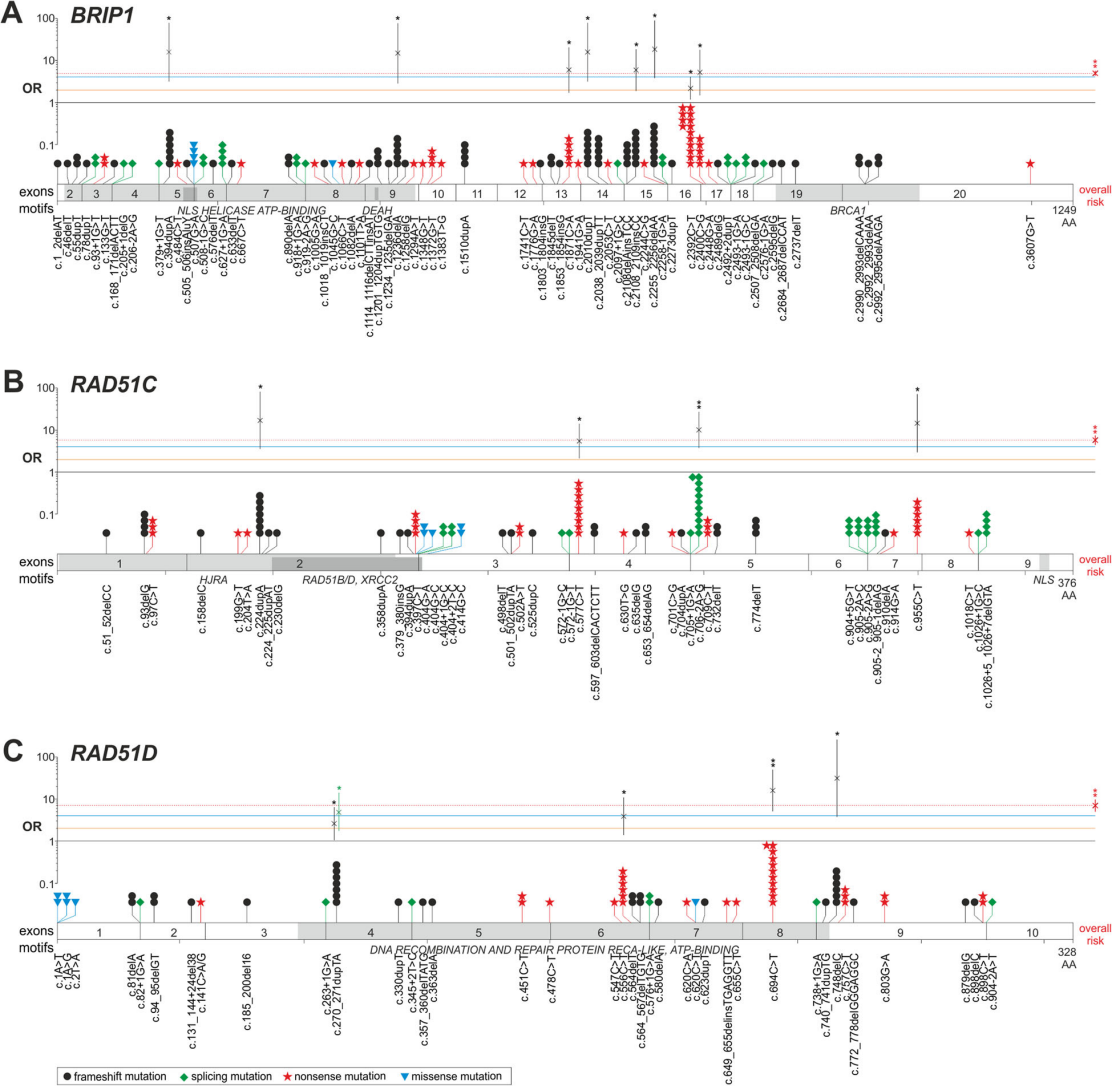
The distribution of *BRIP1* (**a**), *RAD51C* (**b**), and *RAD51D* (**c**) mutations in patients with OC**.** The shape and color of a mutation symbol reflect the type of mutation (see the legend), and the number of symbols reflects the identified number of particular mutations. The structures of the analyzed genes and domains in the corresponding proteins were designed based on the Ensembl genome browser and UniProt database. Horizontal lines highlight an OR of 1 (no risk – black line), an OR of 2 (the threshold for moderate risk – orange line), an OR of 4 (the threshold for high risk – blue line), and the general gene-specific OR established for a particular gene in this study (dashed red line). Gene-specific OR symbols (red x with whiskers indicating 95% CIs) are indicated on the right of each gene chart, and mutation-specific OR symbols are indicated above particular mutation positions. The c.270_271dupTA-specific OR calculated for the East Asian population is indicated in green. * or ** next to the OR symbol indicates a *p*-value < 0.05 and < 0.0001, respectively. Note that the association analysis was performed only for recurrent mutations (≥5 cases) also present in at least one control. The detailed values of ORs for particular mutations in the *BRIP1*, *RAD51C*, and *RAD51D* genes are provided in Table 2

A summary of the studies examining *BRIP1, RAD51C*, and *RAD51D* mutations in OC patients is described in **Table****S1**. The overall mutation prevalence and ORs are presented in Table 1, while the mutation-specific risks for recurrent mutations (*n* ≥ 5) are provided in Table 2. The distribution of mutations alongside the gene sequences with the indicated gene- and mutation-specific ORs are shown in Fig. 1.

**Table 1:** The overall prevalence and association of *BRIP1*, *RAD51C,* and *RAD51D* mutations with OC risk

Gene	M/ALL OC (%)	M/ALL CTR (%)	OR; 95%CIs; *p*	OR_adj_^a^; 95%CIs; *p*
*BRIP1*	200/22494 (0.8891)	209/115375 (0.1811)	4.94; 4.07–6.00; < 0.0001	4.32; 3.48–5.37; < 0.0001
*RAD51C*	149/23802 (0.6260)	130/115475 (0.1126)	5.59; 4.42–7.07; < 0.0001	5.04; 3.85–6.59; < 0.0001
*RAD51D*	94/22787 (0.4125)	72/120688 (0.0597)	6.94; 5.10–9.44; < 0.0001	7.60; 5.29–10.93; < 0.0001

*ALL* total number of cases tested; *CTR* Controls; *M* Number of cases with mutation; *OC* Ovarian cancer cases; ^a^, *OR* Adjusted for the major populations (i.e., Caucasian/European and East Asian)

**Table 2:** The prevalence and association of *BRIP1*, *RAD51C,* and *RAD51D* recurrent mutations (n ≥ 5) with OC risk

Mutation ID (nt level)	Mutation ID (AA level)	M/ALL OC (%)	M/ALL CTR (%)	OR; 95%CIs; *p*
*BRIP1*				
c.394dupA	(p.Thr132Asnfs)	6/22494 (0.0267)	2/118234 (0.0017)	15.77; 3.18–78.15; 0.0007
c.1236delA	(p.Val413Phefs)	5/22494 (0.0222)	2/133990 (0.0015)	14.89; 2.89–76.78; 0.0012
c.1871C > A	(p.Ser624Ter)	5/22494 (0.0222)	5/134109 (0.0037)	5.96; 1.73–20.60; 0.0048
c.2010dupT	(p.Glu671Terfs)	6/22494 (0.0267)	2/118387 (0.0017)	15.79; 3.19–78.25; 0.0007
c.2108_2109insCC	(p.Lys703Asnfs)	6/22494 (0.0267)	6/133895 (0.0045)	5.95; 1.92–18.46; 0.0020
c.2255_2256delAA	(p.Lys752Argfs)	7/22494 (0.0311)	2/118257 (0.0017)	18.41; 3.82–88.61; 0.0003
c.2392C > T	(p.Arg798Ter)	14/22494 (0.0622)	37/131983 (0.0280)	2.22; 1.20–4.11; 0.0110
c.2400C > G	(p.Tyr800Ter)	5/22494 (0.0222)	5/117211 (0.0043)	5.21; 1.51–18.00; 0.0091
*RAD51C*				
c.224dupA	(p.Tyr75Terfs)	7/23802 (0.0294)	2/118461 (0.0017)	17.42; 3.62–83.88; 0.0004
c.577C > T	(p.Arg193Ter)	9/23802 (0.0378)	8/118351 (0.0068)	5.60; 2.16–14.50; 0.0004
c.706-2A > G		11/23802 (0.0462)	6/134110 (0.0045)	10.33; 3.82–27.95; < 0.0001
c.955C > T	(p.Arg319Ter)	6/23802 (0.0252)	2/118382 (0.0017)	14.92; 3.01–73.95; 0.0009
*RAD51D*				
c.270_271dupTA	(p.Lys91Ilefs)	7/22584 (0.0310)	14/118455 (0.0118)	2.62; 1.06–6.50; 0.0373
c.556C > T	(p.Arg186Ter)	6/22584 (0.0266)	9/133163 (0.0068)	3.93; 1.40–11.05; 0.0094
c.694C > T	(p.Arg232Ter)	11/22584 (0.0487)	4/131873 (0.0030)	16.07; 5.12–50.46; < 0.0001
c.748delC	(p.His250Thrfs)	6/22584 (0.0266)	1/118275 (0.0008)	31.43; 3.78–261.09; 0.0014

*ALL* total number of cases tested; *CTR* controls; *M* Number of cases with mutation; *OC* Ovarian cancer cases

### BRIP1

As shown in **Table****S2** in the 44 reviewed studies cumulatively encompassing 22,494 OC cases, we identified 200 *BRIP1* mutations (71 distinct mutations). Most studies (27, n_cases_ = 21,582) were conducted in the Caucasian (or predominantly Caucasian) population, 12 (n_cases_ = 742) in the East Asian population, and 5 (n_cases_ = 170) in other populations. Comparing the mutation frequencies in OC patients (0.89%) and non-cancer controls from the gnomAD database (0.18%), we calculated the cumulative OC risk, OR = 4.94 (95%CIs:4.07–6.00; *p* < 0.0001), and adjusted for major continental-level ethnic groups, OR_adj_ = 4.32 (95%CIs:3.48–5.37; *p* < 0.0001). Studies using older technologies may be less sensitive to detect some variants, therefore we repeated the analysis exclusively with the NGS studies, but the result showed no substantial differences (OR = 4.95; 95%CIs:4.08–6.01; *p* < 0.0001). As shown in Fig. 1a, the *BRIP1* mutations are equally distributed over most of the *BRIP1* coding sequence, with the last exon (exon 20) being much less densely covered. The largest fraction of mutations are frameshift mutations (52%), followed by nonsense mutations (30%), and splicing mutations (15%). Fifteen of the mutations were detected in three or more cases, with the highest occurrence of c.2392C > T (p.Arg798Ter), c.2255_2256delAA (p.Lys752Argfs), c.394dupA (p.Thr132Asnfs), c.2010dupT (p.Glu671Terfs), and c.2108_2109insCC (p.Lys703Asnfs) reported in 14, 7, 6, 6, and 6 cases, respectively. Importantly, all the above mutations were identified only in studies of predominantly Caucasian population. For 8 recurring mutations (identified in at least 5 cases) that have also been reported in controls, we calculated mutation-specific ORs. Despite the low statistical power of this analysis, the mutation-specific ORs confirm that 7 out of 8 recurrent mutations are high-risk variants (ORs > 5). However, it should be noted that the most frequent mutation (p.Arg798Ter) seems to be a medium-risk allele, OR = 2.22 (95%CIs:1.20–4.11; *p* < 0.0001).

### RAD51C

As shown in **Table****S3**, in the 53 selected studies cumulatively encompassing 23,802 OC cases, we identified 149 *RAD51C* mutations (46 distinct mutations). Among the studies, most (35, n_cases_ = 22,862) were of Caucasian individuals, 11 (n_cases_ = 725) were of East Asian individuals, and 7 (n_cases_ = 215) were of individuals from other populations. The prevalence of *RAD51C* mutations was significantly higher in OC patients (0.63%) than in population controls (0.11%), giving a cumulative OR = 5.59 (95%CIs:4.42–7.07; *p* < 0.0001) and OR_adj_ = 5.04 (95%CIs:3.85–6.59; *p* < 0.0001). Limiting the analysis only to the NGS studies did not significantly affect the risk estimates (OR = 5.43; 95%CIs:4.26–6.91; *p* < 0.0001). As shown in Fig. 1b, the distribution of mutations along the coding sequence of *RAD51C* is rather even, however, there was a higher concentration in the middle of the gene. The contribution of different types of mutations is also even, i.e., 31% are frameshift mutations, including 12% duplications and 18% deletions; 31% are nonsense mutations; and 27% are splicing mutations, which proportion in *RAD51C* is significantly enriched in comparison to *BRIP1* (Fisher’s exact test, *p* < 0.05) and *RAD51D* (*p* < 0.05). Fourteen of the mutations were detected in three or more cases, with c.706-2A > G, c.577C > T (p.Arg193Ter), c.224dupA (p.Tyr75Terfs), and c.955C > T (p.Arg319Ter) mutations being the most frequent, reported in 11, 9, 7, and 6 cases, respectively. As in *BRIP1*, the most frequent mutations were exclusively reported in Caucasian studies (or in predominantly Caucasian studies). For 4 recurring mutations also reported in controls, we calculated mutation-specific ORs. The analysis confirmed that all of them, including the most frequent splicing mutation (c.706-2A > G), are associated with a high mutation-specific risk (ORs > 5).

### RAD51D

As shown in **Table****S4**, in the 42 reviewed studies cumulatively encompassing 22,787 OC cases, we identified 94 *RAD51D* mutations (39 distinct mutations). Most (31, n_cases_ = 22,104) studies were performed in the Caucasian (or predominantly Caucasian) population, 8 (n_cases_ = 650) in the East Asian population, and 3 (n_cases_ = 33) in other populations. Comparing the mutation frequency in OC cases (0.41%) and in population controls (0.06%), the cumulative OR = 6.94 (95%CIs:5.10–9.44; *p* < 0.0001) and OR_adj_ = 7.60 (95%CIs:5.29–10.93; *p* < 0.0001) were estimated. Limiting the analysis only to the NGS studies, only slightly reduced the risk estimates (OR = 6.40; 95%CIs:4.65–8.80; *p* < 0.0001). As shown in Fig. 1c, ~ 69% of *RAD51D* mutations are distributed in a part of the gene that corresponds to DNA recombination and repair protein RecA-like, ATP-binding domain of the RAD51D protein. The majority of identified mutations were either frameshift (42%) or nonsense (42%), while splicing alterations accounted for 9% of identified mutations. Five mutations were identified in at least three patients, e.g., c.694C > T (p.Arg232Ter) in 11 cases, c.270_271dupTA (p.Lys91Ilefs) in 7 cases, and c.556C > T (p.Arg186Ter) as well as c.748delC (p.His250Thrfs) in 6 cases each. Three out of 4 recurrent mutations were associated with statistically significant high risk (OR ~ 4 or higher). The exception is the p.Lys91Ilefs mutation, conferring OC moderate risk (OR = 2.62; 95%CIs:1.06–6.50; *p* = 0.04). However, as this mutation was identified predominantly in East Asian patients, we recalculated its OR specifically for the East Asian population. The analysis revealed that the p.Lys91Ilefs mutation is also an OC high-risk allele (OR = 4.89; 95%CIs:1.76–13.62; *p* = 0.0024) specific for the Asian population.

### Large mutations

As most studies did not analyze large mutations, we did not include this type of mutation in the formal calculations of OC risk. Nonetheless, dozens of large mutations (deletions of single or several exons) were reported in the discussed genes in some of the selected studies (Table S5), and they accounted for ~ 7%, ~ 12%, and even ~ 16% of all mutations reported in *BRIP1*, *RAD51C*, and *RAD51D*, respectively. For comparison, no *BRIP1*, 2 *RAD51C*, and 2 *RAD51D* large mutations per ~ 11,000 subjects are reported in the gnomAD database, and 2 *BRIP1*, 5 *RAD51C*, and 3 *RAD51D* large mutations per ~ 10,000 cancer-free women older than age 70 are documented in the FLOSSIES database [92]. These data demonstrate that large mutations account for substantial fractions of deleterious mutations in *BRIP1*, *RAD51C*, and *RAD51D* and therefore may substantially contribute to OC risk.

## Discussion

In contrast to mutations in *BRCA1* and *BRCA2*, which predispose individuals to both BC and OC, *BRIP1*, *RAD51C*, and *RAD51D* are predominantly OC risk genes, while their role in BC is less defined and questionable [20, 21, 27, 74, 75, 93–96]. Despite certain relationships between mutations in the discussed genes and OC, more precise risk estimation is required. For this purpose, large-scale studies and meta-analyses, such as the one presented here, should be beneficial.

To our knowledge, this meta-analysis is the study encompassing the largest number of OC cases to date to estimate *BRIP1*, *RAD51C*, and *RAD51D* mutation prevalence and their contribution to OC risk. Overall, ~ 29,400 OC patients from 63 studies were compared to a total of ~ 116,000 subjects from the gnomAD non-cancer population control database, which serves as a useful reference set of allele frequencies for many genetic studies.

Our results demonstrate that cumulatively 1.93% of OC patients had a mutation in one of the three discussed genes compared with 0.35% in gnomAD non-cancer population controls. Separately, the prevalence of *BRIP1*, *RAD51C*, and *RAD51D* mutations in OC cases was 0.89, 0.63, and 0.41%, respectively. These data are consistent with those indicated in our prior meta-analysis (reported for a 3-7x smaller cohort) [25] and those indicated in the largest study [26], which was included in the current meta-analysis. Summarizing, ~ 2% contribution of *BRIP1*, *RAD51C*, and *RAD51D* mutations in all OC, translates into their 10% contribution in hereditary OC (making up ~ 20% of all OC) [25, 85] which is the largest contribution after *BRCA1*/*2* mutations.

The collection of a large sample size allowed us to achieve higher statistical power and more precisely estimate the overall OC risk attributed to all mutations identified in *BRIP1*, *RAD51C*, and *RAD51D* than in previous studies. The risk estimates fall within the range of ORs from different studies [19, 21, 22, 26, 28, 29, 57], but the confidence intervals obtained in this study were much tighter and the margin of error was smaller. Examples of higher OC risk estimates (OR = ~ 11, and OR = ~ 19) for *BRIP1* mutations were reported [18, 27], but this discrepancy may be due to the varied participation of OC patients with a family history of OC and/or BC and *BRCA1*/*2* mutation carrier exclusion in several studies, as well as modest sample sizes.

The practical interpretation of the functional significance of missense variants is problematic and requires experimental confirmation or co-segregation of mutation and the disease within the family, as the predicted computational importance may be far from the actual clinical phenotype [97]. Therefore, we included in our estimates only missense variants that are classified as pathogenic/likely pathogenic in the ClinVar database or those that were shown experimentally by authors to be deleterious, such as the c.507G > A mutation (rs876660937) within the *BRIP1* gene [27]. The mutation was detected in several OC and BC patients, but not in population controls, and caused the skipping of exon 5, resulting in a shorter BRIP1 product [27]. We also included two *RAD51C* intron variants (found in BC and OC cases), i.e., c.904 + 5G > T, and c.1026 + 5_1026 + 7del, located close to the splicing sites. These mutations were shown to disrupt the *RAD51C* mRNA splicing and are considered to be likely pathogenic variants [20, 98]. Large mutations were not included in risk estimates, however, it is suggested that they may contribute to familial breast/ovarian cancer risk [99, 100]. As mentioned in the Results section, large mutations constitute a substantial fraction of all reported mutations, especially in the *RAD51C* and *RAD51D* genes; therefore, they may also influence the risk of OC. Indeed, a duplication encompassing most of the *RAD51C* gene (exons 1–7) was identified in several patients with OC and BC in Finland, but not in population controls [101], confirming our observations.

Considering the generally dispersed distribution of mutations in *BRIP1*, *RAD51C*, and *RAD51D*, diagnostic sequencing of the entire coding sequences of the genes is recommended. Nevertheless, some observations about the distribution of mutations are worth mentioning. Firstly, a lower occurrence of mutations in *BRIP1* exon 20 is noticeable, which may result from their lower predicted functional consequences, and therefore fewer mutations reported in this region. Secondly, there is an accumulation of mutations in the central part of *RAD51D* (DNA recombination and repair protein RecA-like, ATP-binding domain), however, due to the still limited number of mutations, this hotspot and their potential association with OC phenotype may not be precisely recognized. Finally, significantly more mutations occur in splice sites in *RAD51C*, than in other analyzed genes.

Taking advantage of the large scale of the analysis, we were able to identify recurrent, potentially founder mutations that were difficult to recognize in smaller studies. In proportion to the analyzed populations, most recurrent mutations were detected in the Caucasian (or predominantly Caucasian) studies, with the exception of one mutation characteristic for the East Asian population.

We aimed to determine the mutation-specific risk associated with specific mutations recurring in OC cases, as some mutations within a particular gene may be associated with a lower or higher risk than the general risk estimated for the gene. Examples of varied risks associated with specific mutations within the same gene have been previously reported in case-control genotyping studies. For example, the c.7271 T > G (p.Val2424Gly) and c.1036C > T (p.Arg346Cys) mutations in the BC moderate-risk genes *ATM* and *CHEK2*, respectively, individually conferred a high risk of BC [102]. Additionally, a strong association for the common variant (rs17507066) in *CHEK2* with OC was shown [103], while the overall OC risk attributed to *CHEK2* mutations is low (OR ~ 1) [25, 26]. Mutation-specific cancer risks and the existence of either BC and OC cluster regions have also been reported before for *BRCA1*/*2* [104–107]. In our study, most recurrent mutations were associated with a high risk of OC. The exceptions were two alleles conferring a moderate risk, i.e., the *BRIP1* p.Arg798Ter and *RAD51D* p.Lys91Ilefs mutations. However, the risk estimate for the latter mutation is most likely biased, as *RAD51D* p.Lys91Ilefs is predominantly an East Asian mutation. The calculation of OR specifically for the East Asian population confirmed the high risk of OC attributed to the p.Lys91Ilefs mutation. The *BRIP1* p.Arg798Ter and *RAD51D* p.Lys91Ilefs mutations were also shown to be a moderate-risk allele for prostate cancer [108] and BC [109], respectively. Consistent with our results, two Finnish recurrent mutations (c.93delG and c.837 + 1G > A) in *RAD51C* and a founder mutation (c.576 + 1G > A) in *RAD51D* were shown to be OC high-risk mutations [19, 22].

The findings reported in the current analysis should be considered in light of certain limitations that could have affected the risk estimates. First, the studies qualified for the analysis may differ, starting with patients’ inclusion/exclusion criteria, through the methodology used, and ending with variant calling and classification. For example, to collect the maximum number of OC patients, the histological subtype of OC was not taken into account. As different histological subtypes of OC represent distinct disease entities, the mutation prevalence may be underestimated, especially that HR deficiency is primarily associated with high-grade serous OC. Also, no minimum sequencing quality/coverage was defined for the inclusion of the studies and the mutation calling. As this information is rarely included, low-coverage studies, with reduced ability to detect some mutation types (e.g., insertions and deletions) may be included in our analysis, underestimating the true mutation prevalence. Second, due to the lack of control groups in most of the studies, to calculate mutation-associated risks (ORs), we used unmatched gnomAD non-cancer controls. Although when calculating the mutation-associated risks, we took into account the major (continental-level) human populations, there still may be some differences in population stratification between cases and controls that may affect the results. Third, as the study is limited mainly to the Caucasian population, the findings may not represent the other populations with different mutation distributions. Therefore, to better understand genetic susceptibility to OC in different populations, additional large-scale studies in such populations, are needed. Fourth, although we have made every effort to remove the results of duplicated samples, individual cases of such duplicates may still exist in our data and may affect the results, potentially causing false recurrence of some mutation. Fifth, regardless of a large number of OC cases included in this study, the analysis of mutation-specific risk was of relatively low statistical power and statistically significant ORs could be provided only for the most frequent mutations. Thus, even more extensive studies and consortia collecting data from different studies are needed. Until then, the general risk associated with all mutations will be used, supported with information from in silico prediction tools and functional assays for specific mutations. Sixth, the risk estimates presented in our study, as well as in other studies, are almost exclusively based on loss-of-function mutations due to difficulties associated with interpreting the consequences of missense or in-frame variants, not to mention the non-protein-coding intronic or regulatory variants. Therefore, we cannot rule out that we missed potentially pathogenic variants (especially those defined currently as variants of unknown significance). Finally, although most of the results obtained in our study were confirmed by appropriate tests with very low *p*-values, it has to be noted that, some of the borderline-significant results should be interpreted with consideration of the number of performed tests.

## Conclusions

The findings of our study add to previous pieces of evidence that *BRIP1*, *RAD51C*, and *RAD51D* mutations contribute to the development of OC. The mutation prevalence and OC risk estimates (including recurrent mutation-specific risk) for mutation carriers provided here support the interpretation of diagnostic results and personalized genetic counseling and highlight the need for routine *BRIP1*, *RAD51C*, and *RAD51D* gene analysis in OC high-risk patients (e.g., first-degree relatives of women with OC). As mutations are generally dispersed in the entire genes, sequencing of entire coding sequences of the genes is recommended. Interestingly, a lower prevalence of *BRIP1* mutations in its last exon, a higher proportion of splicing mutations in *RAD51C*, and possible hotspot in *RAD51D* are observed. Future studies are also required, with much larger sample sizes, stratified by both OC tumor types and ethnicity groups, to define the risk more precisely, and for a larger set of specific mutations, as well as to understand the impact of other factors on risk modification in mutation carriers.

## Supplementary information

**Additional file 1: Figure S1.** The flow diagram indicating the strategy and criteria used for the selection of articles for the analysis. **Table S1.** Characteristics of the studies eligible for meta-analysis. **Table S2.** The list of *BRIP1* mutations identified in patients with OC, with their prevalence in OC patients and population controls and associated mutation-specific OC risk. **Table S3.** The list of *RAD51C* mutations identified in patients with OC, with their prevalence in OC patients and population controls and associated mutation-specific OC risk. **Table S4.** The list of *RAD51D* mutations identified in patients with OC, with their prevalence in OC patients and population controls and associated mutation-specific OC risk. **Table S5.** The list of *BRIP1*, *RAD51C*, and *RAD51D* large mutations reported in studies selected for analysis.

## Data Availability

All data generated or analyzed during this study are included in this published article and its supplementary information files.

## Abbreviations

BC: Breast cancer
CIs: Confidence intervals
HR: Homologous recombination
NGS: Next-generation sequencing
OC: Ovarian cancer
OR: Odds ratio
PARP: Poly-ADP-ribose polymerase
