# Supplementary material for: BRIP1, RAD51C, and RAD51D mutations are associated with high susceptibility to ovarian cancer: mutation prevalence and precise risk estimates based on a pooled analysis of ~30,000 cases
Source: J Ovarian Res. 2020 May 2;13:50. doi: 10.1186/s13048-020-00654-3 (PMC7196220; doi:10.1186/s13048-020-00654-3)
Supplement: Supplementary file 1 — Additional file 1: Figure S1. The flow diagram indicating the strategy and criteria used for the selection of articles for the analysis. Table S1. Characteristics of the studies eligible for meta-analysis. Table S2. The list of BRIP1 mutations identified in patients with OC, with their prevalence in OC patients and population controls and associated mutation-specific OC risk. Table S3. The list of RAD51C mutations identified in patients with OC, with their prevalence in OC patients and population controls and associated mutation-specific OC risk. Table S4. The list of RAD51D mutations identified in patients with OC, with their prevalence in OC patients and population controls and associated mutation-specific OC risk. Table S5. The list of BRIP1, RAD51C, and RAD51D large mutations reported in studies selected for analysis. [file 13048_2020_654_MOESM1_ESM.docx]

**Figure**


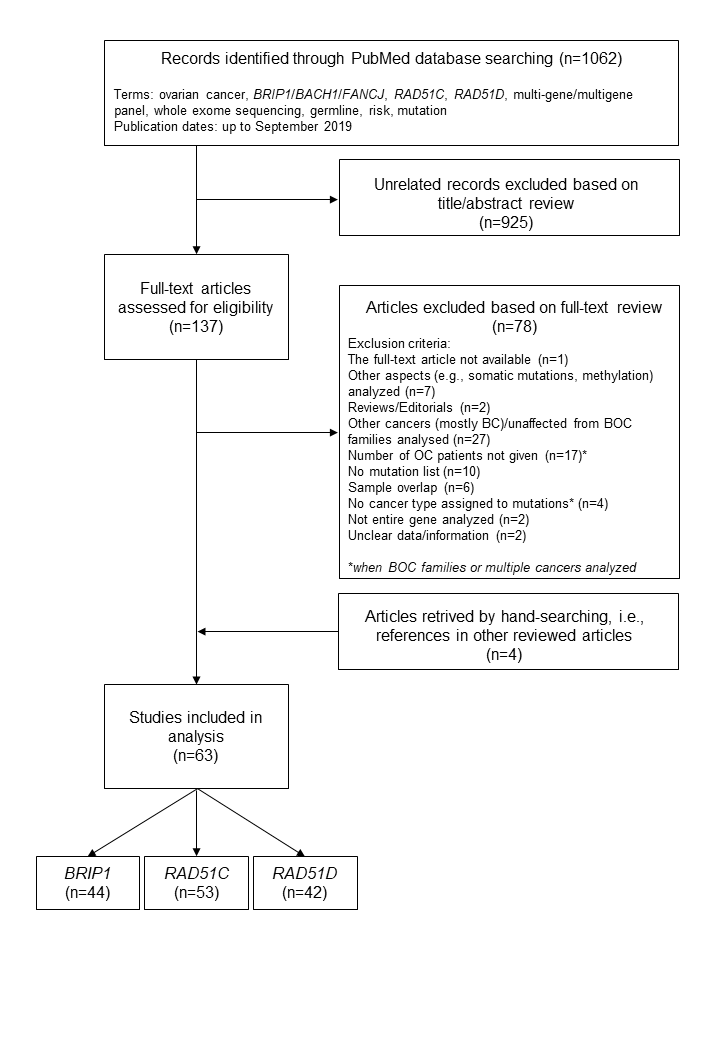


**Figure S1.** The flow diagram indicating the strategy and criteria used for the selection of articles for the analysis.

**Tables**

**Table S1. Characteristics of the studies eligible for meta-analysis.**

| **Study, Year** | **Country/Predominant ethnicity** | **M P/LP** | **Case No.^1^** | **Method** | **Family history of cancers^2^** | ***BRCA1/2* status** |
| --- | --- | --- | --- | --- | --- | --- |
| ***BRIP1*** | | | | | | |
| Lilyquist, 2017 | USA/Mixed (Caucasian:76%) | 71 | 8226 | NGS | 86% FHC, 45% FHBC, 15% FHOC | **-** |
| Carter, 2018 | USA/Mixed (Caucasian:75%) | 34 | 4439 | NGS | 19% FHOC | **-** |
| Ramus, 2015 | 10 studies: UK, USA, Australia, Germany, Belarus/Caucasian | 30 | 3257 | NGS | 19% FHBOC, 13% FHBC, 9% FHOC | - |
| Norquist, 2016* | USA/Mixed (Caucasian:88%) | 23 | 1915 | NGS | Unselected (% not provided) | - |
| Susswein, 2015 | USA/Mixed (Caucasian:82%) | 6 | 845 | NGS | % not provided | - |
| Weber-Lassalle , 2018 | Germany/Caucasian | 19 | 706 | NGS | 60% FHBC, 27% FHOC | *BRCA1/2*neg |
| Harter, 2017 | Germany/Caucasian (97%) | 2 | 523 | NGS | 43% FHBOC | - |
| Dicks, 2017** | The Cancer Genome Atlas cases (UK, USA, Australia, Scotland, Germany, Belarus)/Mixed (Caucasian:86%) | 3 | 412 | WES/NGS | % not provided | - |
| Koczkowska, 2018 | Poland/Caucasian | 1 | 333 | NGS | Unselected (% not provided) | - |
| Feliubadaló, 2019 | Spain/Caucasian | 2 | 262 | NGS | % not provided | - |
| Krivokuca, 2019 | Serbia/Caucasian | 2 | 131 | NGS | 21% FHC, 19% FHBOC | - |
| Eoh, 2017 | South Korea/Asian | 1 | 117 | NGS | 55% FHC | - |
| Shirts, 2016 | USA/- | 1 | 112 | NGS | 44% FHC | - |
| Ow, 2019 | Singapore/Mixed (Chinese: 60%) | 1 | 102 | NGS | 53% FHBC, 20% FHOC | - |
| Frey, 2017*** | USA/Mixed (Caucasian:80%) | 1 | 49 | NGS | 70% FHBC, 14% FHOC | - |
| Mannan, 2016 | India/Asian | 1 | 17 | NGS | 75% FHB0C | - |
| Teer, 2017 | USA/- | 1 | 9 | WES/NGS | % not provided | - |
| Sung, 2017 | Taiwan/Asian | 1 | 7 | NGS | 15% FHBOC | - |
| Hirasawa, 2017 | Japan/Asian | 0 | 230 | NGS | 14% FHBC, 7% FHOC | - |
| Siraj, 2017 | Saudi Arabia/Arabs | 0 | 117 | NGS | 7% FHBOC | - |
| Crawford, 2017 | USA/Mixed (Caucasian:66%) | 0 | 99 | NGS | % not provided | *BRCA1/2*neg |
| Hirotsu, 2015 | Japan/Asian | 0 | 79 | NGS | 94% FHC | *BRCA1/2*neg  (checked 93% of patients) |
| Li, 2019 | China/Asian | 0 | 62 | NGS | Unselected (16% FHBOC) | - |
| Zhao, 2017 | China/Asian | 0 | 50 | NGS | Unselected (22% FHC) | - |
| Stafford, 2017 | USA/Caucasian | 0 | 48 | WES/NGS | 65% FHBC, 29% FHOC | *BRCA1/2*neg |
| Tsaousis, 2019 | Greece, Romania, Turkey/Mixed | 0 | 42 | NGS | 84% FHC | - |
| Bonache, 2018 | Spain/Caucasian | 0 | 38 | NGS | 100% FHBOC | *BRCA1/2*neg |
| Bernards, 2016* | USA/- | 0 | 36 | NGS | Unselected (36% strong FHBOC) | - |
| Tedaldi, 2017 | Italy/Caucasian | 0 | 36 | NGS | 81% FHBOC | - |
| Chirasophon, 2017 | Thailand/Asian | 0 | 35 | NGS | 4% strong FHBOC | - |
| Bertelsen, 2019 | Denmark/Caucasian | 0 | 23 | WES/NGS | % not provided | - |
| Catucci, 2012 | Italy, Israel/Ashkenazim | 0 | 22 | DGGE or HRM, then S | 100% FHBOC | *BRCA1/2*neg^3^ |
| Schubert, 2019 | Germany/Caucasian | 0 | 20 | NGS | 100% FHC, 33% strong FHBOC | *BRCA1/2*neg |
| Kanke, 2018 | Japan/Asian | 0 | 19 | WES/NGS | Unselected (% not provided) | - |
| Wong, 2016 | Singapore/Asian | 0 | 18 | NGS | 87% FHBC, 13% FHOC | - |
| Yablonski-Peretz, 2016 | Israel/Mixed (Ashkenazim:53% ) | 0 | 15 | NGS | 100% FHC | - |
| Byers, 2016 | UK/Caucasian | 0 | 10 | NGS | 100% FHBOC | *BRCA1/2*neg |
| Zidan, 2017 | Israel/Arabs | 0 | 9 | NGS | 100% FHBOC | - |
| Cock-Rada, 2017 | Colombia/Latino | 0 | 7 | NGS | Criteria for HBOC genetic testing (NCCN) | - |
| Kuusisto , 2011 | Finland/Caucasian | 0 | 6 | S | 17% FHBOC | *BRCA1/2*neg^3^ |
| Sato, 2017 | Japan/Asian | 0 | 6 | S | Criteria for BRCA1/2 testing (NCCN) | *BRCA1/2*neg |
| Maksimenko, 2018 | Latvia/Caucasian | 0 | 2 | NGS | Criteria for BRCA1/2 testing (NCCN) | *BRCA1/2*neg^3^ |
| Wong, 2011 | Australia, Sweden/Caucasian | 0 | 2 | S | 100% FHBOC | *BRCA1/2*neg |
| Feliubadalo, 2017 | Spain/Caucasian | 0 | 1 | WES/NGS | 100% FHC | *BRCA1/2*neg |
| ***RAD51C*** | | | | | | |
| Lilyquist, 2017 | USA/Mixed (Caucasian:76%) | 55 | 8226 | NGS | 86% FHC, 45% FHBC, 15% FHOC | - |
| Carter, 2018 | USA/Mixed (Caucasian:75%) | 28 | 4439 | NGS | 19% FHOC | - |
| Song, 2015 | 8 cohorts: UK, Australia, USA, Denmark, Poland/Caucasian | 13 | 3429 | NGS | 14% FHBC, 13% FHOC | - |
| Norquist, 2016* | USA/Mixed (Caucasian:88%) | 9 | 1915 | NGS | Unselected (% not provided) | - |
| Susswein, 2015 | USA/Mixed (Caucasian:82%) | 5 | 845 | NGS | % not provided | - |
| Loveday, 2012 | UK/Caucasian (97%) | 7 | 721 | S | 62% FHBOC, 38% Unselected (% not provided) | *BRCA1/2*neg  (familial cohotr) |
| Harter, 2017 | Germany/Caucasian (97%) | 10 | 523 | NGS | 43% FHBOC | - |
| Dicks, 2017** | The Cancer Genome Atlas cases (UK, USA, Australia, Scotland, Germany, Belarus)/Mixed (Caucasian:86%) | 2 | 412 | WES/NGS | % not provided | - |
| Koczkowska, 2018 | Poland/Caucasian | 1 | 333 | NGS | Unselected (% not provided) | - |
| Thompson, 2011 | Australia/Caucasian | 1 | 267 | HRM, then S | 44% Unselected (% not provided) | - |
| Feliubadaló, 2019 | Spain/Caucasian | 1 | 262 | NGS | % not provided | - |
| Vuorela, 2011 | Finland, Sweden/Caucasian | 1 | 232 | HRM, then S | Unselected (% not provided) | - |
| Janatova, 2015 | Czech Republic/Caucasian | 2 | 171 | HRM, then S | 36% FHBOC | *BRCA1/2*neg |
| Krivokuca, 2019 | Serbia/Caucasian | 2 | 131 | NGS | 19% FHBOC | - |
| Eoh, 2017 | South Korea/Asian | 2 | 117 | NGS | 55% FHC | - |
| Siraj, 2017 | Saudi Arabia/Arabs | 1 | 117 | NGS | 7% FHBOC | - |
| Ow, 2019 | Singapore/Mixed (Chinese: 60%) | 1 | 102 | NGS | 53% FHBC, 20% FHOC | - |
| Crawford, 2017 | USA/Mixed (Caucasian:66%) | 1 | 99 | NGS | % not provided | *BRCA1/2*neg |
| Coulet, 2013 | France/Caucasian | 2 | 69 | S | 49% FHBC, 12% FHOC | *BRCA1/2*neg |
| Li, 2019 | China/Asian | 1 | 62 | NGS | Unselected (16% FHBOC) | - |
| Blanco, 2014 | Spain/Caucasian | 1 | 58 | S | 100% FHBOC | *BRCA1/2*neg |
| Sánchez-Bermúdez, 2018 | Spain/Caucasian | 1 | 43 | S | 100% FHBOC | *BRCA1/2*neg |
| Chirasophon, 2017 | Thailand/Asian | 1 | 35 | NGS | 4% strong FHBOC | - |
| Rashid, 2014 | Pakistan, Germany, USA/Asian | 1 | 34 | DHPLC, then S | 35% FHBOC | *BRCA1/2*neg |
| Hirasawa, 2017 | Japan/Asian | 0 | 230 | NGS | 14% FHBC, 7% FHOC | - |
| Clague, 2011 | USA/Mixed (Caucasian:58%) | 0 | 153 | S | 100% FHBOC | *BRCA1/2*neg |
| Leeneer, 2012 | Belgium, Canada, The Netherlands, UK/Caucasian | 0 | 124 | S or HRM, then S | 100% FHBOC | *BRCA1/2*neg |
| Shirts, 2016 | USA/- | 0 | 112 | NGS | 44% FHC | - |
| Hirotsu, 2015 | Japan/Asian | 0 | 79 | NGS | 94% FHC | *BRCA1/2*neg  (checked 93% of patients) |
| Zhao, 2017 | China/Asian | 0 | 50 | NGS | Unselected (22% FHC) | - |
| Stafford, 2017 | USA/Caucasian | 0 | 48 | WES/NGS | 65% FHBC, 29% FHOC | *BRCA1/2*neg |
| Tsaousis, 2019 | Greece, Romania, Turkey/Mixed | 0 | 42 | NGS | 84% FHC | - |
| Bonache, 2018 | Spain/Caucasian | 0 | 38 | NGS | 100% FHBOC | *BRCA1/2*neg |
| Bernards, 2016* | USA/- | 0 | 36 | NGS | Unselected (36% strong FHBOC) | - |
| Tedaldi, 2017 | Italy/Caucasian | 0 | 36 | NGS | 81% FHBOC | - |
| Bertelsen, 2019 | Denmark/Caucasian | 0 | 23 | WES/NGS | % not provided | - |
| Zheng, 2010 | USA/Mixed (Caucasian:82%) | 0 | 22 | S | 100% FHBOC | *BRCA1/2*neg |
| Schubert, 2019 | Germany/Caucasian | 0 | 20 | NGS | 100% FHC, 33% FHBOC | *BRCA1/2*neg |
| Kanke, 2018 | Japan/Asian | 0 | 19 | WES/NGS | Unselected (% not provided) | - |
| Wong, 2016 | Singapore/Asian | 0 | 18 | NGS | 87% FHBC, 13% FHOC | - |
| Mannan, 2016 | India/Asian | 0 | 17 | NGS | 75% FHB0C | - |
| Kushnir, 2012 | Israel/Mixed (Ashkenazi 38%) | 0 | 16 | S | 100% FHBOC | *BRCA1/2*neg^3^ |
| Yablonski-Peretz, 2016 | Israel/Mixed (Ashkenazim:53% ) | 0 | 15 | NGS | 100% FHC | - |
| Byers, 2016 | UK/Caucasian | 0 | 10 | NGS | 100% FHBOC | *BRCA1/2*neg |
| Lu, 2012 | USA, China/Caucasian | 0 | 9 | HRM, then S | 100% FHBOC | *BRCA1/2*neg |
| Teer, 2017 | USA/- | 0 | 9 | WES/NGS | % not provided | - |
| Zidan, 2017 | Israel/Arabs | 0 | 9 | NGS | 100% FHBOC | - |
| Cock-Rada, 2017 | Colombia/Latino | 0 | 7 | NGS | Criteria for HBOC genetic testing (NCCN) | - |
| Sung, 2017 | Taiwan/Asian | 0 | 7 | NGS | 15% FHBOC | - |
| Sato, 2017 | Japan/Asian | 0 | 6 | S | Criteria for BRCA1/2 testing (NCCN) | *BRCA1/2*neg |
| Maksimenko, 2018 | Latvia/Caucasian | 0 | 2 | NGS | Criteria for BRCA1/2 testing (NCCN) | *BRCA1/2*neg^3^ |
| Wong, 2011 | Australia, Sweden/Caucasian | 0 | 2 | S | 100% FHBOC | *BRCA1/2*neg |
| Feliubadalo, 2017 | Spain/Caucasian | 0 | 1 | WES/NGS | 100% FHC | *BRCA1/2*neg |
| ***RAD51D*** | | | | | | |
| Lilyquist, 2017 | USA/Mixed (Caucasian:76%) | 24 | 7537 | NGS | 86% FHC, 45% FHBC, 15% FHOC | **-** |
| Carter, 2018 | USA/Mixed (Caucasian:75%) | 14 | 4439 | NGS | 19% FHOC | **-** |
| Song, 2015 | 8 cohorts: UK, Australia, USA, Denmark, Poland/Caucasian | 12 | 3429 | NGS | 14% FHBC, 13% FHOC | - |
| Norquist, 2016* | USA/Mixed (Caucasian:88%) | 9 | 1915 | NGS | Unselected (% not provided) | - |
| Susswein, 2015 | USA/Mixed (Caucasian:82%) | 3 | 845 | NGS | % not provided | - |
| Konstanta, 2018 | Greece/Caucasian | 1 | 609 | S | Unselected (20% FHBOC) | *BRCA1/2*neg |
| Harter, 2017 | Germany/Caucasian (97%) | 3 | 523 | NGS | 43% FHBOC | - |
| Dicks, 2017** | The Cancer Genome Atlas cases (UK, USA, Australia, Scotland, Germany, Belarus)/Mixed (Caucasian:86%) | 2 | 412 | WES/NGS | % not provided | - |
| Thompson, 2013 | Australia, UK/Caucasian | 2 | 343 | HRM, then S | 29% FHBOC, 71% Unselected (% not provided) | *BRCA1/2*neg  (familial cohort) |
| Loveday, 2011 | UK/Caucasian (97%) | 4 | 271 | S | 100% FHBOC | *BRCA1/2*neg |
| Feliubadaló, 2019 | Spain/Caucasian | 1 | 262 | NGS | % not provided | - |
| Gutiérrez-Enríquez, 2014 | Spain/Caucasian | 3 | 239 | HRM or DHPLC or S, then S | 73% FHBOC | *BRCA1/2*neg |
| Hirasawa, 2017 | Japan/Asian | 3 | 230 | NGS | 14% FHBC, 7% FHOC | - |
| Janatova, 2015 | Czech Republic/Caucasian | 3 | 171 | HRM, then S | 36% FHBOC | *BRCA1/2*neg |
| Eoh, 2017 | South Korea/Asian | 2 | 117 | NGS | 55% FHC | - |
| Shirts, 2016 | USA/- | 1 | 112 | NGS | 44% FHC | - |
| Ow, 2019 | Singapore/Mixed (Chinese: 60%) | 1 | 102 | NGS | 53% FHBC, 20% FHOC | - |
| Stafford, 2017 | USA/Caucasian | 2 | 48 | WES/NGS | 65% FHBC, 29% FHOC | *BRCA1/2*neg |
| Sánchez-Bermúdez, 2018 | Spain/Caucasian | 1 | 42 | S | 100% FHBOC | *BRCA1/2*neg |
| Bonache, 2018 | Spain/Caucasian | 2 | 38 | NGS | 100% FHBOC | *BRCA1/2*neg |
| Tedaldi, 2017 | Italy/Caucasian | 1 | 36 | NGS | 81% FHBOC | - |
| Koczkowska, 2018 | Poland/Caucasian | 0 | 333 | NGS | Unselected (% not provided) | - |
| Krivokuca, 2019 | Serbia/Caucasian | 0 | 131 | NGS | 19% FHBOC | - |
| Crawford, 2017 | USA/Mixed (Caucasian:66%) | 0 | 99 | NGS | % not provided | *BRCA1/2*neg |
| Hirotsu, 2015 | Japan/Asian | 0 | 79 | NGS | 94% FHC | *BRCA1/2*neg  (checked 93% of patients) |
| Osher, 2012 | Canada, Belgium/Caucasian | 0 | 78 | S or HRM, then S | 100% FHBOC | *BRCA1/2*neg |
| Zhao, 2017 | China/Asian | 0 | 50 | NGS | Unselected (22% with FHC) | - |
| Frey, 2017*** | USA/Mixed (Caucasian:80%) | 0 | 49 | NGS | 70% FHBC, 14% FHOC | - |
| Tsaousis, 2019 | Greece, Romania, Turkey/Mixed | 0 | 42 | NGS | 84% FHC | - |
| Bernards, 2016* | USA/- | 0 | 36 | NGS | Unselected, 36% strong FHBOC | - |
| Chirasophon, 2017 | Thailand/Asian | 0 | 35 | NGS | 4% strong FHBOC | - |
| Bertelsen, 2019 | Denmark/Caucasian | 0 | 23 | WES/NGS | % not provided | - |
| Schubert, 2019 | Germany/Caucasian | 0 | 20 | NGS | 100% FHC, 33% strong FHBOC | *BRCA1/2*neg |
| Kanke, 2018 | Japan/Asian | 0 | 19 | WES/NGS | Unselected (% not provided) | - |
| Wong, 2016 | Singapore/Asian | 0 | 18 | NGS | 87% FHBC, 13% FHOC | - |
| Mannan, 2016 | India/Asian | 0 | 17 | NGS | 75% FHB0C | - |
| Byers, 2016 | UK/Caucasian | 0 | 10 | NGS | 100% FHBOC | *BRCA1/2*neg |
| Zidan, 2017 | Israel/Arabs | 0 | 9 | NGS | 100% FHBOC | - |
| Teer, 2017 | USA/- | 0 | 9 | WES/NGS | % not provided | - |
| Cock-Rada, 2017 | Colombia/Latino | 0 | 7 | NGS | Criteria for HBOC genetic testing (NCCN) | - |
| Maksimenko, 2018 | Latvia/Caucasian | 0 | 2 | NGS | Criteria for BRCA1/2 testing (NCCN) | *BRCA1/2*neg^3^ |
| Feliubadalo, 2017 | Spain/Caucasian | 0 | 1 | WES/NGS | 100% FHC | *BRCA1/2*neg |

^1^, Only patients with a personal history of OC were included (OC patients were extracted from familial BC and/or OC studies); ^2^, % of patients with a family history (FH) of breast cancer (BC), ovarian cancer (OC), breast/ovarian cancers (BOC) or all cancers (C); ^3^, Negative for selected BRCA1/2 founder mutations; "*", indicates studies, including the same sample sets - studies in brackets were not used in the OR calculation; DGGE, Denaturing gradient gel electrophoresis; DHPLC, Denaturing high-performance liquid chromatography; HRM, High-resolution melting; M P/LP, Number of pathogenic/likely pathogenic mutations; NGS, Next-generation sequencing; S, Sequencing; WES, Whole-exome sequencing

**Table S2. The list of *BRIP1* mutations identified in patients with OC, with their prevalence in OC patients and population controls and associated mutation-specific OC risk.**

| Mutation ID (nt level) | Mutation ID (AA level) | Type/Subtype | dbSNP | M^#^/ALL OC (%) | M/ALL CTR (%) | OR; 95%CIs; *p* | Reported in |
| --- | --- | --- | --- | --- | --- | --- | --- |
| c.1_2delAT | (p.Met1Valfs) | FS_DEL/START_ | rs876661246 | 1/22494 (0.0044) | 0/- (nd) |  | Carter |
| c.46delT | (p.Tyr16Thrfs) | FS_DEL_ | rs876660613 | 1/22494 (0.0044) | 0/- (nd) |  | Lilyquist |
| c.55dupT | (p.Tyr19Leufs) | FS_DUP_ | - | 2/22494 (0.0089) | 0/- (nd) |  | Ramus, Weber-Lassalle |
| c.78dupT | (p.Ala27Cysfs) | FS_DUP_ | rs1555618709 | 1/22494 (0.0044) | 0/- (nd) |  | Ramus |
| c.93+1G>T |  | S | rs587782047 | 2/22494 (0.0089) | 0/- (nd) |  | Ramus, Norquist |
| c.133G>T | (p.Glu45Ter) | N | rs587781292 | 2/22494 (0.0089) | 1/134135 (0.0007) |  | Lilyquist, Ramus |
| c.168_171delACTT | (p.Leu56Phefs) | FS_DEL_ | - | 1/22494 (0.0044) | 0/- (nd) |  | Ramus |
| c.205+1delG |  | S | rs1057517648 | 1/22494 (0.0044) | 0/- (nd) |  | Lilyquist |
| c.206-2A>G |  | S | rs786203700 | 1/22494 (0.0044) | 1/118080 (0.0008) |  | Norquist |
| c.379+1G>T |  | S | - | 1/22494 (0.0044) | 0/- (nd) |  | Ramus |
| c.394dupA | (p.Thr132Asnfs) | FS_DUP_ | rs587781416 | 6/22494 (0.0267) | 2/118234 (0.0017) | 15.77; 3.18-78.15; 0.0007 | Lilyquist, Ramus, Norquist |
| c.484C>T | (p.Arg162Ter) | N | rs747604569 | 1/22494 (0.0044) | 2/118392 (0.0017) |  | Feliubadaló |
| c.505_506insAluY | (p.Gln169Argfs) | FS_IN_ | - | 1/22494 (0.0044) | 0/- (nd) |  | Ow |
| c.507G>A | (p.Gln169Gln)/(p.Ser128Ter)* | MS/S* | rs876660937 | 4/22494 (0.0178) | 0/- (nd) |  | Weber-Lassalle |
| c.508-1G>C |  | S | rs864622277 | 2/22494 (0.0089) | 0/- (nd) |  | Lilyquist, Norquist |
| c.576delT | (p.Val193Terfs) | FS_DEL&N_ | rs876660769 | 1/22494 (0.0044) | 0/- (nd) |  | Lilyquist |
| c.627+1G>A |  | S | rs587780833 | 4/22494 (0.0178) | 0/- (nd) |  | Lilyquist, Carter, Susswein, Weber-Lassalle |
| c.633delT | (p.Gly212Alafs) | FS_DEL_ | rs779466229 | 1/22494 (0.0044) | 4/117410 (0.0034) |  | Mannan |
| c.667C>T | (p.Gln223Ter) | N | rs786201733 | 1/22494 (0.0044) | 0/- (nd) |  | Lilyquist |
| c.890delA | (p.Lys297Serfs) | FS_DEL_ | rs786202610 | 2/22494 (0.0089) | 0/- (nd) |  | Lilyquist, Norquist |
| c.918+1G>A |  | S | rs587781655 | 2/22494 (0.0089) | 4/118059 (0.0034) |  | Lilyquist, Ramus |
| c.919-2A>G |  | S | - | 1/22494 (0.0044) | 0/- (nd) |  | Ramus |
| c.1005G>A | (p.Trp335Ter) | N | rs786201808 | 1/22494 (0.0044) | 0/- (nd) |  | Lilyquist |
| c.1018_1019insCT | (p.Leu340Profs) | FS_IN_ | rs878855134 | 1/22494 (0.0044) | 0/- (nd) |  | Lilyquist |
| c.1045G>C | (p.Ala349Pro) | MS | rs149364097 | 1/22494 (0.0044) | 4/134094 (0.0030) |  | Carter, [Lu] |
| c.1066C>T | (p.Arg356Ter) | N | rs730881633 | 1/22494 (0.0044) | 3/134089 (0.0022) |  | Lilyquist |
| c.1082delA | (p.Asp361Valfs) | FS_DEL_ | rs587781639 | 1/22494 (0.0044) | 0/- (nd) |  | Lilyquist |
| c.1101T>A | (p.Cys367Ter) | N | - | 1/22494 (0.0044) | 0/- (nd) |  | Weber-Lassalle |
| c.1114_1116delCTTinsAT | (p.Leu372Ilefs) | FS_INDEL_ | rs587783377 | 1/22494 (0.0044) | 0/- (nd) |  | Lilyquist |
| c.1201_1204dupTGTG | (p.Ala402Valfs) | FS_DUP_ | rs730881647 | 3/22494 (0.0133) | 0/- (nd) |  | Lilyquist, Carter, Susswein |
| c.1234_1235delGA | (p.Glu412Serfs) | FS_DEL_ | rs1064795649 | 1/22494 (0.0044) | 0/- (nd) |  | Lilyquist |
| c.1236delA | (p.Val413Phefs) | FS_DEL_ | rs863224525 | 5/22494 (0.0222) | 2/133990 (0.0015) | 14.89; 2.89-76.78; 0.0012 | Lilyquist, Ramus, Norquist, Weber-Lassalle, Dicks |
| c.1258delG | (p.Asp420Metfs) | FS_DEL_ | - | 1/22494 (0.0044) | 0/- (nd) |  | Norquist |
| c.1294A>T | (p.Lys432Ter) | N | rs1555607070 | 1/22494 (0.0044) | 0/- (nd) |  | Teer |
| c.1348G>T | (p.Glu450Ter) | N | - | 1/22494 (0.0044) | 0/- (nd) |  | Ramus |
| c.1372G>T | (p.Glu458Ter) | N | rs587780228 | 3/22494 (0.0133) | 3/132502 (0.0023) |  | Carter, Norquist |
| c.1383T>G | (p.Tyr461Ter) | N | rs587780875 | 1/22494 (0.0044) | 0/- (nd) |  | Weber-Lassalle |
| c.1510dupA | (p.Ile504Asnfs) | FS_DUP_ | rs775735278 | 4/22494 (0.0178) | 1/118415 (0.0008) |  | Lilyquist, Ramus, Norquist, Koczkowska |
| c.1741C>T | (p.Arg581Ter) | N | rs780020495 | 1/22494 (0.0044) | 5/118455 (0.0042) |  | Harter |
| c.1776G>A | (p.Trp592Ter) | N | rs753023295 | 1/22494 (0.0044) | 0/- (nd) |  | Eoh |
| c.1803_1804insG | (p.Asp602Glyfs) | FS_IN_ | - | 1/22494 (0.0044) | 0/- (nd) |  | Weber-Lassalle |
| c.1845delT | (p.Thr616Hisfs) | FS_DEL_ | - | 2/22494 (0.0089) | 0/- (nd) |  | Ramus |
| c.1853_1854insG | (p.Pro619Thrfs) | FS_IN_ | rs587781985 | 1/22494 (0.0044) | 1/118464 (0.0008) |  | Lilyquist |
| c.1871C>A | (p.Ser624Ter) | N | rs587781321 | 5/22494 (0.0222) | 5/134109 (0.0037) | 5.96; 1.73-20.60; 0.0048 | Lilyquist, Carter, Ramus, Norquist, [Lu] |
| c.1941G>A | (p.Trp647Ter) | N | rs786202760 | 1/22494 (0.0044) | 0/- (nd) |  | Lilyquist |
| c.2010dupT | (p.Glu671Terfs) | FS_DUP&N_ | rs775537066 | 6/22494 (0.0267) | 2/118387 (0.0017) | 15.79; 3.19-78.25; 0.0007 | Lilyquist, Carter, Ramus, Norquist, Weber-Lassalle, [Pennington], [Walsh] |
| c.2038_2039dupTT | (p.Leu680Phefs) | FS_DUP_ | rs587778134 | 5/22494 (0.0222) | 0/- (nd) |  | Lilyquist, Carter, Ramus, Norquist |
| c.2053C>T | (p.Gln685Ter) | N | rs876659533 | 1/22494 (0.0044) | 2/15695 (0.0127) |  | Weber-Lassalle |
| c.2097+1G>C |  | S | rs786202941 | 1/22494 (0.0044) | 0/- (nd) |  | Lilyquist |
| c.2108delAinsTCC | (p.Lys703Ilefs) | FS_INDEL_ | rs786203384 | 4/22494 (0.0178) | 0/- (nd) |  | Lilyquist, Carter, Norquist, Susswein, [Lu], [Pennington], [Walsh] |
| c.2108_2109insCC | (p.Lys703Asnfs) | FS_IN_ | - | 6/22494 (0.0267) | 6/133895 (0.0045) | 5.95; 1.92-18.46; 0.0020 | Ramus, Norquist, Dicks |
| c.2244C>G | (p.Tyr748Ter) | N | - | 1/22494 (0.0044) | 0/- (nd) |  | Sung |
| c.2255_2256delAA | (p.Lys752Argfs) | FS_DEL_ | rs730881649 | 7/22494 (0.0311) | 2/118257 (0.0017) | 18.41; 3.82-88.61; 0.0003 | Lilyquist, Carter, Ramus, Susswein, Weber-Lassalle |
| c.2258-1G>A |  | S | rs1064793887 | 2/22494 (0.0089) | 0/- (nd) |  | Carter, Harter |
| c.2273dupT | (p.Ala759Serfs) | FS_DUP_ | rs587780236 | 1/22494 (0.0044) | 1/118339 (0.0008) |  | Weber-Lassalle |
| c.2392C>T | (p.Arg798Ter) | N | rs137852986 | 14/22494 (0.0622) | 37/131983 (0.0280) | 2.22; 1.20-4.11; 0.0110 | Lilyquist, Carter, Ramus, Norquist, Susswein, Weber-Lassalle, [Pennington], Feliubadaló, Krivokuca, Shirts, Frey |
| c.2400C>G | (p.Tyr800Ter) | N | rs574552037 | 5/22494 (0.0222) | 5/117211 (0.0043) | 5.21; 1.51-18.00; 0.0091 | Lilyquist, Carter, Norquist, Weber-Lassalle |
| c.2448G>A | (p.Trp816Ter) | N | rs1064795352 | 1/22494 (0.0044) | 0/- (nd) |  | Carter |
| c.2489delG | (p.Gly830Valfs) | FS_DEL_ | - | 1/22494 (0.0044) | 0/- (nd) |  | Dicks |
| c.2492+2dupT |  | S | rs587780240 | 2/22494 (0.0089) | 0/- (nd) |  | Lilyquist, Carter |
| c.2493-1G>A |  | S | rs786203451 | 1/22494 (0.0044) | 1/118251 (0.0008) |  | Carter |
| c.2493-1G>C |  | S | rs786203451 | 2/22494 (0.0089) | 0/- (nd) |  | Ramus, Krivokuca |
| c.2507_2508delGA | (p.Arg836Lysfs) | FS_DEL_ | - | 1/22494 (0.0044) | 0/- (nd) |  | Ramus |
| c.2576-1G>A |  | S | rs587782539 | 1/22494 (0.0044) | 0/- (nd) |  | Lilyquist |
| c.2595delG | (p.Gln866Serfs) | FS_DEL_ | rs587781974 | 1/22494 (0.0044) | 0/- (nd) |  | Lilyquist |
| c.2684_2687delCCAT | (p.Ser895Terfs) | FS_DUP&N_ | rs760551339 | 1/22494 (0.0044) | 4/134087 (0.0030) |  | Lilyquist |
| c.2737delT | (p.Ser913Leufs) | FS_DEL_ | - | 1/22494 (0.0044) | 0/- (nd) |  | Lilyquist |
| c.2990_2993delCAAA | (p.Thr997Argfs) | FS_DEL_ | rs771028677 | 2/22494 (0.0089) | 8/118224 (0.0068) |  | Lilyquist,Carter |
| c.2992_2993delAA | (p.Lys998Glufs) | FS_DEL_ | rs878855151 | 1/22494 (0.0044) | 1/118224 (0.0008) |  | Lilyquist |
| c.2992_2995delAAGA | (p.Lys998Glufs) | FS_DEL_ | rs786203717 | 2/22494 (0.0089) | 5/133968 (0.0037) |  | Carter, Weber-Lassalle |
| c.3607G>T | (p.Glu1203Ter) | N | - | 1/22494 (0.0044) | 0/- (nd) |  | Ramus |

*, As reported in Weber-Lassalle et al.; M, number of cases with mutation; ^#^, the exact numbers of OC cases carrying a particular mutation were not available in two studies [26, 85], therefore we treated each individual mutation from these studies as a single variant in the calculations; ALL, total number of cases tested; OC, ovarian cancer cases; CTR, controls; FS_DEL_, a frameshift mutation – deletion; FS_DEL/START_, a frameshift mutation – deletion, interrupting the first codon; FS_DEL&N_ – a frameshift mutation – deletion, immediately leading to a codon stop; FS_DUP_, a frameshift mutation – duplication; FS_DUP&N_, a frameshift mutation – duplication, immediately leading to a codon stop; FS_IN_, a frameshift mutation – insertion; FS_INDEL_, a frameshift mutation – insertion and deletion; MS, a missense mutation; N, a nonsense mutation; S, a splice site mutation

**Table S3. The list of *RAD51C* mutations identified in patients with OC, with their prevalence in OC patients and population controls and associated mutation-specific OC risk.**

| Mutation ID (nt level) | Mutation ID (AA level) | Type/Subtype | dbSNP | M^#^/ALL OC (%) | M/ALL CTR (F%) | OR; 95%CIs; *p* | Reported in |
| --- | --- | --- | --- | --- | --- | --- | --- |
| c.51_52delCC | (p.Pro18Alafs) | FS_DEL_ | - | 1/23802 (0.0042) | 0/- (nd) |  | Siraj |
| c.93delG | (p.Phe32Serfs) | FS_DEL_ | rs730881942 | 4/23802 (0.0168) | 13/134168 (0.0097) |  | Lilyquist, Carter, Norquist, Susswein |
| c.97C>T | (p.Gln33Ter) | N | rs587782528 | 3/23802 (0.0126) | 3/118471 (0.0025) |  | Lilyquist, Carter, Song |
| c.158delC | (p.Ser53Leufs) | FS_DEL_ | rs587782533 | 1/23802 (0.0042) | 0/- (nd) |  | Lilyquist |
| c.199G>T | (p.Glu67Ter) | N |  | 1/23802 (0.0042) | 0/- (nd) |  | Harter |
| c.204T>A | (p.Cys68Ter) | N | - | 1/23802 (0.0042) | 0/- (nd) |  | Rashid |
| c.224dupA | (p.Tyr75Terfs) | FS_DUP_ | rs730881939 | 7/23802 (0.0294) | 2/118461 (0.0017) | 17.42; 3.62-83.88; 0.0004 | Lilyquist, Norquist, Harter, [Pennington], [Walsh] |
| c.224_225dupAT | (p.Ala76Metfs) | FS_DUP_ | - | 1/23802 (0.0042) | 0/- (nd) |  | Lilyquist |
| c.230delG | (p.Gly77Valfs) | FS_DEL_ | rs1057519355 | 1/23802 (0.0042) | 0/- (nd) |  | Thompson |
| c.358dupA | (p.Thr120Asnfs) | FS_DUP_ | - | 1/23802 (0.0042) | 0/- (nd) |  | Feliubadaló |
| c.379_380insG | (p.Pro127Argfs) | FS_IN_ | - | 1/23802 (0.0042) | 0/- (nd) |  | Janatova |
| c.394dupA | (p.Thr132Asnfs) | FS_DUP_ | rs730881940 | 1/23802 (0.0042) | 10/132750 (0.0075) |  | Lilyquist |
| c.397C>T | (p.Gln133Ter) | N | rs387907159 | 4/23802 (0.0168) | 0/- (nd) |  | Lilyquist, Carter, Loveday, Crawford |
| c.404G>A | (p.Cys135Tyr) | MS | rs767796996 | 2/23802 (0.0084) | 1/116464 (0.0009) |  | Lilyquist, Sánchez-Bermúdez |
| c.404G>C | (p.Cys135Ser) | MS | rs767796996 | 1/23802 (0.0042) | 0/- (nd) |  | Carter |
| c.404+1G>C |  | S | - | 2/23802 (0.0084) | 0/- (nd) |  | Krivokuca |
| c.404+2T>C |  | S | rs730881931 | 2/23802 (0.0084) | 1/116197 (0.0009) |  | Carter, Susswein |
| c.414G>C | (p.Leu138Phe) | MS | rs267606999 | 2/23802 (0.0084) | 1/118469 (0.0008) |  | Lilyquist, Carter |
| c.498delT | (p.Asp167Ilefs) | FS_DEL_ | rs746993675 | 1/23802 (0.0042) | 1/118467 (0.0008) |  | Song |
| c.501_502dupTA | (p.Arg168Ilefs) | FS_DUP_ | rs587782699 | 1/23802 (0.0042) | 0/- (nd) |  | Lilyquist |
| c.502A>T | (p.Arg168Ter) | N | rs587781490 | 2/23802 (0.0084) | 1/118470 (0.0008) |  | Lilyquist, Harter |
| c.525dupC | (p.Cys176Leufs) | FS_DUP_ | rs768793789 | 1/23802 (0.0042) | 1/118465 (0.0008) |  | Harter |
| c.572-1G>C |  | S | rs1413872299 | 1/23802 (0.0042) | 0/- (nd) |  | Lilyquist |
| c.572-1G>T |  | S | - | 1/23802 (0.0042) | 1/118359 (0.0008) |  | Song |
| c.577C>T | (p.Arg193Ter) | N | rs200293302 | 9/23802 (0.0378) | 8/118351 (0.0068) | 5.60; 2.16-14.50; 0.0004 | Lilyquist, Carter, Song, Norquist, Susswein, Loveday, [Lu], Dicks, [Pennington], Coulet |
| c.597_603delCACTCTT | (p.Phe199Leufs) | FS_DEL_ | - | 2/23802 (0.0084) | 0/- (nd) |  | Eoh |
| c.630T>G | (p.Tyr210Ter) | N | rs786201909 | 1/23802 (0.0042) | 0/- (nd) |  | Lilyquist |
| c.635delG | (p.Arg212Profs) | FS_DEL_ | - | 1/23802 (0.0042) | 0/- (nd) |  | Li |
| c.653_654delAG | (p.Glu218Valfs) | FS_DEL_ | rs587782286 | 2/23802 (0.0084) | 0/- (nd) |  | Lilyquist, Song |
| c.701C>G | (p.Ser234Ter) | N | rs587782818 | 1/23802 (0.0042) | 16/118293 (0.0135) |  | Lilyquist |
| c.704dupA | (p.Val236Glyfs) | FS_DUP_ | - | 1/23802 (0.0042) | 0/- (nd) |  | Loveday |
| c.705+1G>A |  | S | rs876658644 | 1/23802 (0.0042) | 0/- (nd) |  | Norquist |
| c.706-2A>G |  | S | rs587780259 | 11/23802 (0.0462) | 6/134110 (0.0045) | 10.33; 3.82-27.95; <0.0001 | Lilyquist, Carter, Song, Norquist, Susswein, Loveday, Harter, [Pennington], [Walsh], Koczkowska |
| c.709C>T | (p.Arg237Ter) | N | rs770637624 | 3/23802 (0.0126) | 3/118423 (0.0025) |  | Lilyquist, Carter, Blanco |
| c.732delT | (p.Ile244Metfs) | FS_DEL_ | rs1060502601 | 2/23802 (0.0084) | 0/- (nd) |  | Song, Norquist |
| c.774delT | (p.Thr259Leufs) | FS_DEL_ | rs754367349 | 3/23802 (0.0126) | 11/118450 (0.0093) |  | Carter, Song, Vuorela |
| c.904+5G>T |  | IVS | rs587782702 | 3/23802 (0.0126) | 4/118055 (0.0034) |  | Lilyquist, Carter, Loveday |
| c.905-2A>C |  | S | rs779582317 | 3/23802 (0.0126) | 4/134100 (0.0030) |  | Norquist, Ow, Chirasophon |
| c.905-2A>G |  | S | rs779582317 | 3/23802 (0.0126) | 0/- (nd) |  | Lilyquist, Carter, Coulet |
| c.905-2_905-1delAG |  | S | rs587781995 | 4/23802 (0.0168) | 3/118401 (0.0025) |  | Lilyquist, Carter, Song, [Lu], Dicks |
| c.910delA | (p.Ser304Valfs) | FS_DEL_ | rs1555603011 | 1/23802 (0.0042) | 0/- (nd) |  | Carter |
| c.914G>A | (p.Trp305Ter) | N | rs876659874 | 1/23802 (0.0042) | 1/15695 (0.0064) |  | Carter |
| c.955C>T | (p.Arg319Ter) | N | rs587781287 | 6/23802 (0.0252) | 2/118382 (0.0017) | 14.92; 3.01-73.95; 0.0009 | Lilyquist, Carter, Song, Norquist, Loveday |
| c.1018C>T | (p.Gln340Ter) | N | rs1555605103 | 1/23802 (0.0042) | 0/- (nd) |  | Lilyquist |
| c.1026+1G>C |  | S | - | 1/23802 (0.0042) | 0/- (nd) |  | Harter |
| c.1026+5_1026+7delGTA |  | IVS | rs587781410 | 4/23802 (0.0168) | 1/118428 (0.0008) |  | Lilyquist, Carter, Susswein, Janatova |

M, number of cases with mutation; ^#^, the exact numbers of OC cases carrying a particular mutation were not available in two studies [26, 85], therefore we treated each individual mutation from these studies as a single variant in the calculations; ALL, total number of cases tested; OC, ovarian cancer cases; CTR, controls; FS_DEL_, a frameshift mutation – deletion; FS_DUP_, a frameshift mutation – duplication; FS_IN_, a frameshift mutation – insertion; IVS, an intron mutation; MS, a missense mutation; N, a nonsense mutation; S, a splice site mutation

**Table S4. The list of *RAD51D* mutations identified in patients with OC, with their prevalence in OC patients and population controls and associated mutation-specific OC risk.**

| Mutation ID (nt level) | Mutation ID (AA level) | Type/Subtype | dbSNP | M^#^/ALL OC (%) | M/ALL CTR (F%) | OR; 95%CIs; *p* | Reported in |
| --- | --- | --- | --- | --- | --- | --- | --- |
| c.1A>T | (p.Met1Leu) | MS_START_ | rs561425038 | 2/22584 (0.0089) | 0/- (nd) |  | Carter, Gutiérrez-Enríquez |
| c.1A>G | (p.Met1Val) | MS_START_ | rs561425038 | 2/22584 (0.0089) | 0/- (nd) |  | Carter, Susswein |
| c.2T>A | (p.Met1Lys) | MS_START_ | rs1064794619 | 1/22584 (0.0044) | 0/- (nd) |  | Carter |
| c.81delA | (p.Val28Trpfs) | FS_DEL_ | rs1064793952 | 2/22584 (0.0089) | 0/- (nd) |  | Carter, Norquist |
| c.82+1G>A |  | S | rs786202788 | 1/22584 (0.0044) | 0/- (nd) |  | Lilyquist |
| c.94_95delGT | (p.Val32Phefs) | FS_DEL_ | rs786203137 | 2/22584 (0.0089) | 1/118471 (0.0008) |  | Lilyquist,Bonache |
| c.131_144+24del38 |  | FS_INDEL_ | rs1064795716 | 1/22584 (0.0044) | 1/118449 (0.0008) |  | Norquist, [Pennington], [Wickramanayake] |
| c.141C>A/G | (p.Tyr47Ter) | N | - | 1/22584 (0.0044) | 1/118465 (0.0008) |  | Norquist, [Pennington] |
| c.185_200del16 | (p.Ser62Leufs) | FS_DEL_ | rs786202179 | 1/22584 (0.0044) | 0/- (nd) |  | Lilyquist |
| c.263+1G>A |  | S | rs1555570242 | 1/22584 (0.0044) | 0/- (nd) |  | Norquist |
| c.270_271dupTA | (p.Lys91Ilefs) | FS_DUP_ | rs753862052 | 7/22584 (0.0310) | 14/118455 (0.0118) | 2.62; 1.06-6.50; 0.0373 | Lilyquist, Loveday, Hirasawa, Eoh, Ow |
| c.330dupT | (p.Ser111Terfs) | FS_DUP_ | rs786202434 | 1/22584 (0.0044) | 0/- (nd) |  | Lilyquist |
| c.345+2T>C |  | S | rs876659394 | 1/22584 (0.0044) | 0/- (nd) |  | Lilyquist |
| c.357_360delTATG | (p.Cys119Trpfs) | FS_DEL_ | rs876658297 | 1/22584 (0.0044) | 0/- (nd) |  | Harter |
| c.363delA | (p.Ala122Glnfs) | FS_DEL_ | rs730881935 | 1/22584 (0.0044) | 4/118442 (0.0034) |  | Lilyquist |
| c.451C>T | (p.Gln151Ter) | N | rs587781756 | 2/22584 (0.0089) | 1/118463 (0.0008) |  | Norquist, Stafford |
| c.478C>T | (p.Gln160Ter) | N | rs1057521922 | 1/22584 (0.0044) | 0/- (nd) |  | Song |
| c.547C>T | (p.Gln183Ter) | N | rs587782695 | 1/22584 (0.0044) | 0/- (nd) |  | Lilyquist |
| c.556C>T | (p.Arg186Ter) | N | rs387906843 | 6/22584 (0.0266) | 9/133163 (0.0068) | 3.93; 1.40-11.05; 0.0094 | Lilyquist, [Lu], Dicks, Thompson, Loveday, Stafford |
| c.564delT | (p.Val189Trpfs) | FS_DEL_ | rs786202750 | 2/22584 (0.0089) | 0/- (nd) |  | Lilyquist, Song |
| c.564_567delTGTG | (p.Val189Profs) | FS_DEL_ | - | 2/22584 (0.0089) | 0/- (nd) |  | Song |
| c.576+1G>A |  | S | rs781161543 | 2/22584 (0.0089) | 2/132964 (0.0015) |  | Song, Harter |
| c.580delA | (p.Thr194Leufs) | FS_DEL_ | - | 1/22584 (0.0044) | 0/- (nd) |  | Norquist, [Pennington], [Wickramanayake] |
| c.620C>A | (p.Ser207Ter) | N | - | 1/22584 (0.0044) | 0/- (nd) |  | Song |
| c.620C>T | (p.Ser207Leu) | MS | rs370228071 | 1/22584 (0.0044) | 0/- (nd) |  | Shirts |
| c.623dupT | (p.Thr209Hisfs) | FS_DUP_ | rs1555567610 | 1/22584 (0.0044) | 0/- (nd) |  | Song |
| c.649_655delinsTGAGGTT | (p.Gly217Ter) | N | rs587781527 | 1/22584 (0.0044) | 0/- (nd) |  | Feliubadaló |
| c.655C>T | (p.Gln219Ter) | N | rs771007945 | 1/22584 (0.0044) | 2/118450 (0.0017) |  | Song |
| c.694C>T | (p.Arg232Ter) | N | rs587780104 | 11/22584 (0.0487) | 4/131873 (0.0030) | 16.07; 5.12-50.46; <0.0001 | Lilyquist, Carter, Norquist, Harter, [Pennington], [Wickramanayake], Gutiérrez-Enríquez, Janatova, Sánchez-Bermúdez, Bonache, Tedaldi |
| c.738+1G>A |  | S | - | 1/22584 (0.0044) | 0/- (nd) |  | Konstanta |
| c.740_741dupTG | (p.Thr248Terfs) | FS_DUP&N_ | rs1555567197 | 1/22584 (0.0044) | 0/- (nd) |  | Song |
| c.748delC | (p.His250Thrfs) | FS_DEL_ | rs587780105 | 6/22584 (0.0266) | 1/118275 (0.0008) | 31.43; 3.78-261.09; 0.0014 | Lilyquist, Carter, Song, Susswein |
| c.757C>T | (p.Arg253Ter) | N | rs137886232 | 3/22584 (0.0133) | 4/118312 (0.0034) |  | Norquist, Loveday |
| c.772_778delGGGAGGC | (p.Gly258Serfs) | FS_DEL_ | rs1064795045 | 1/22584 (0.0044) | 0/- (nd) |  | Carter |
| c.803G>A | (p.Trp268Ter) | N | rs750219200 | 2/22584 (0.0089) | 1/118457 (0.0008) |  | Thompson, Loveday |
| c.879delG | (p.Cys294Valfs) | FS_DEL_ | - | 1/22584 (0.0044) | 0/- (nd) |  | Janatova |
| c.898delC | (p.Arg300Aspfs) | FS_DEL_ | rs786202251 | 1/22584 (0.0044) | 0/- (nd) |  | Lilyquist |
| c.898C>T | (p.Arg300Ter) | N | rs750621215 | 2/22584 (0.0089) | 7/118420 (0.0059) |  | Carter, Song |
| c.904-2A>T |  | S | rs1403784434 | 1/22584 (0.0044) | 1/118475 (0.0008) |  | Eoh |

M, number of cases with mutation; ^#^, the exact numbers of OC cases carrying a particular mutation were not available in two studies [26, 85], therefore we treated each individual mutation from these studies as a single variant in the calculations; ALL, total number of cases tested; OC, ovarian cancer cases; CTR, controls; FS_DEL_, a frameshift mutation – deletion; FS_DUP_, a frameshift mutation – duplication; FS_DUP&N_, a frameshift mutation – duplication, immediately leading to a codon stop; FS_INDEL_, a frameshift mutation – insertion and deletion; MS, a missense mutation; MS_START_, a missense mutation, interrupting the first codon; N, a nonsense mutation; S, a splice site mutation

**Table S5. The list of *BRIP1*, *RAD51C*, and *RAD51D* large mutations reported in studies selected for analysis.**

| Mutation ID | Type/Subtype | Reported in |
| --- | --- | --- |
| *BRIP1* | | |
| EX1_2del | L_DEL_ | Lilyquist |
| EX1_4del | L_DEL_ | Carter |
| EX8del | L_DEL_ | Carter |
| EX8_9del | L_DEL_ | Lilyquist |
| EX10_12del | L_DEL_ | Lilyquist |
| EX11-13del | L_DEL_ | Carter, Norquist |
| EX14del | L_DEL_ | Lilyquist |
| EX15del | L_DEL_ | Ramus, Norquist |
| EX17_18del | L_DEL_ | Carter |
| EX18del | L_DEL_ | Norquist |
| *RAD51C* | | |
| 5'UTR_EX4del | L_DEL_ | Lilyquist |
| EX3del | L_DEL_ | Lilyquist |
| EX3del(partial) | L_DEL_ | Lilyquist |
| EX4del | L_DEL_ | Lilyquist |
| EX4_7,9del | L_DEL_ | Norquist |
| c.706-?_837+? EX5del | L_DEL_ | Harter |
| c.706-4423_1131+7851 EX5_9del | L_DEL_ | Harter |
| EX5_9del | L_DEL_ | Lilyquist, Carter |
| EX5_3'UTRdel | L_DEL_ | Lilyquist |
| EX7del | L_DEL_ | Lilyquist |
| EX8del | L_DEL_ | Lilyquist |
| EX8_In8del | L_DEL_ | Lilyquist |
| *RAD51D* | | |
| 5'UTR_EX3del | L_DEL_ | Lilyquist |
| EX1_3del | L_DEL_ | Norquist |
| EX1_8del | L_DEL_ | Carter |
| EX2_in6del | L_DEL_ | Lilyquist |
| EX3del | L_DEL_ | Lilyquist, Carter, Susswein |
| EX9_10del | L_DEL_ | Lilyquist |
| EX10del | L_DEL_ | Norquist |
| In9_3'UTRdel | L_DEL_ | Lilyquist |

L_DEL_, large mutation – deletion
